# Understanding Nephrotic Syndrome Using Kidney Transcriptome Profiling and Computational Studies

**DOI:** 10.34067/KID.0000000000000117

**Published:** 2023-04-27

**Authors:** Paulina X. Medina Rangel, Xuefei Tian

**Affiliations:** Section of Nephrology, Yale University School of Medicine, New Haven, Connecticut

**Keywords:** nephrotic syndrome, transcriptional profiling

Nephrotic syndrome (NS) is a major clinical manifestation of glomerular diseases in children and adult patients that mainly affects the podocytes. Despite decades of research in NS, the underlying causes are still not fully understood, and effective therapeutic agents that promote complete remission are still limited.^1^ Thanks to computational studies and machine learning, kidney transcriptome profiling is evolving at an impressive rate and has great potential to provide new insights into the pathogenesis and mechanisms of kidney diseases from a molecular, cellular, and clinical point of view.^2^ Genome scale expression datasets, such as the Nephrotic Syndrome Study Network (NEPTUNE) and the European Renal cDNA Bank, encompass clinical, genetic, transcriptional, proteomic, and metabolomic information.^3,4^ Therefore, in the last decade, they have become valuable resources for nephrologists and researchers seeking to identify genes and mechanisms whose expression is related to kidney disorders.

In this issue, Li *et al.* performed several computational analyses of a NEPTUNE Study database (GSE200828) to identify shared molecular pathways across patients with FSGS, membranous nephropathy (MN), and minimal change disease (MCD).^5^ FSGS, MN, and MCD are distinct forms of glomerular diseases that all manifest NS. The identification of common molecular pathways in NS will potentially establish known and novel mechanisms of podocyte injury that may translate into the development of novel therapeutic targets to arrest proteinuria and preserve kidney function.

To attain the goal, the authors first identified glomerular differentially expressed genes (DEGs) in FSGS, MN, and MCD and compared with the healthy kidney by using GEO2R, an interactive web tool that allows users to compare and identify DEGs among two or more groups of samples. Next, R packages and machine learning were used to identify enriched pathways in the Kyoto Encyclopedia of Genes and Genomes database. At last, the authors implemented the Cytoscape, Database for Annotation, Visualization and Integrated Discovery (DAVID) Bioinformatic, and STRING tools to reveal the most significant shared pathways contributing to FSGS, MN, and MCD.

Their results suggest that these three types of NS share more than a thousand DEGs. The enrichment analysis identified focal adhesion as the most significative shared dysregulated pathway and established zyxin (*ZYX*) as the most important contributor to these glomerulopathies, followed by actinin 4 (*ACTN4*), *CRK*, and integrins (*ITGA*). Finally, to determine which type of glomerular cells express these genes, the authors used a single-cell RNA-seq dataset, obtained from glomerular-enriched cells in normal kidney tissue from a patient who underwent nephrectomy. Single-cell RNA-seq was processed with the Leiden algorithm from the Phyton Scanpy package. The findings revealed that *ZYX*, *ACTN4*, *CRK*, and *ITGA3* are expressed in most known kidney cell types, especially podocytes.

These results are of great interest because they confirmed, through bioinformatics analysis, the knowledge that we have acquired through clinical and experimental data: Proteinuric kidney diseases are the result of damage to the glomerular filtration barrier, and podocyte injury plays a central role in the initiation and progression of glomerular diseases. They also strengthen the evidence that dysregulation of focal adhesion is a key pathway in these glomerulopathies. In fact, in the past 20 years, numerous studies, including our investigation, have associated proteinuria with dysregulated actin dynamics and altered cell signaling at focal adhesion sites on podocytes.^6,7^ These disorders are manifested by foot process effacement and podocyte depletion. Hence, therapeutic agents that target focal adhesion and actin cytoskeleton on podocytes have been postulated in numerous studies as potential treatments for proteinuric kidney diseases.

Another interesting finding reported by Li *et al.* is the identification of zyxin as the central hub in the FSGS, MN, and MCD. In 2010, different research groups reported that zyxin mediates important mechanisms for the maintenance and repair of the actin cytoskeleton in different epithelial cell lines.^8,9^ In 2013, Tan and coworkers studied the function of zyxin in an immortalized human podocyte cell line. Their findings suggested that zyxin plays a key role in podocyte structure and function using Nef interaction and that zyxin expression is increased to repair actin stress fibers and maintain podocyte phenotype despite the loss of cytoskeletal integrity.^10^ Therefore, it is not surprising that computational studies identified *ZYX* among the most dysregulated genes in NS. However, altered zyxin expression may only be a consequence of the disease state, rather than being responsible for the development of the NS.

A limitation of the study presented by Li *et al.* is the lack of experimental data that validates their bioinformatic findings. Certainly, future directions for this research will be (*1*) to confirm whether zyxin expression in glomerular podocytes in patients with NS is increased, (*2*) to correlate zyxin expression with disease status and progression, and (*3*) to create strategies that modulate zyxin function on kidney injury. Studies investigating zyxin as a potential therapeutic target will be highly valuable.

Although it is very useful to identify shared pathways for proteinuric kidney diseases, several investigations have also attempted to determine, at the cellular level, the distinctive dysregulated pathway in each glomerular ailment. Sealfon *et al.* applied a machine learning framework to determine the molecular characterization of primary MN that distinguished it from FSGS and MCD. The authors found that MN-specific genes were enriched in the nuclear factor kappa B signaling pathway and were predominantly expressed in podocytes.^11^ In a similar study, Zambrano *et al.* performed single-cell sequencing and observed that endothelial cells played an important role in the recruitment of immune cells in the pathogenesis of IgA nephropathy.^12^ Furthermore, urinary single-cell transcriptomics have revealed that urine samples from patients with FSGS contains increased numbers of immune cells and a higher expression of epithelial–mesenchymal transition-associated genes in numerous kidney epithelial cells when compared with the MCD counterparts.^13^ The presence of all kidney cell types and immune cells in urine has also been demonstrated by single-cell transcriptomic analysis in patients with diabetic kidney disease.^14^

As illustrated in the previous paragraphs, the immense potential of kidney transcriptomics and bioinformatics has attracted researchers and nephrologists seeking to identify shared and distinct dysregulated molecular pathways in proteinuric kidney diseases. During the last decade, kidney transcriptomics using human and mouse models has become progressively more feasible and accessible to the scientific community. To take full advantage of these rich datasets, efficient gene sequencing and computational methods are required. Hence, we recommend following guidelines developed by experts, such as the one published by Luecken and Theis,^15^ where they provide workflow recommendations throughout the entire gene sequencing and raw data processing (quality control, quantification, normalization, clustering, identification and validation of DEGs, etc.). Making use of these guidelines will have a substantial positive effect on the outcome.

In sum, kidney transcriptomics has been increasingly used to understand genome-wide transcriptomic variations in different conditions such as disease states. Tissue (bulk) RNA-seq, widely used in human patients and animal models, generates an output for the average expression of genes, reflected by cell type–specific gene expression differences and changes in cell composition. The emerging technology, single-cell RNA-seq, has enabled cell type–specific transcriptome profiling and identified the specific cell types involved in disease development and progression. However, its application in clinical studies still has some limitations to overcome, such as high cost and issues with cell dissociation in solid tissues. Combining tissue RNA-seq with single-cell profiling methods, as Li *et al.* performed in this issue, is a promising and more approachable strategy for understanding cell type–specific and cell heterogeneity changes in NS. The knowledge gained will greatly facilitate future research and clinical work.

## References

[1] Medina Rangel PX Priyadarshini A Tian X. New insights into the immunity and podocyte in glomerular health and disease: from pathogenesis to therapy in proteinuric kidney disease. Integr Med Nephrol Androl. 2021;8(1):5. doi:10.4103/imna.imna_26_21

[2] Kopp JB Anders HJ Susztak K, . Podocytopathies. Nat Rev Dis Primers. 2020;6(1):68. doi:10.1038/s41572-020-0196-7 32792490 PMC8162925

[3] Schmid H Cohen CD Henger A Irrgang S Schlondorff D Kretzler M. Validation of endogenous controls for gene expression analysis in microdissected human renal biopsies. Kidney Int. 2003;64(1):356–360. doi:10.1046/j.1523-1755.2003.00074.x 12787429

[4] Gadegbeku CA Gipson DS Holzman LB, . Design of the Nephrotic Syndrome Study Network (NEPTUNE) to evaluate primary glomerular nephropathy by a multidisciplinary approach. Kidney Int. 2013;83(4):749–756. doi:10.1038/ki.2012.428 23325076 PMC3612359

[5] Li D Liu L Murea M Freedman BI Ma L. Bioinformatics analysis reveals a shared pathway for common forms of adult nephrotic syndrome. Kidney360. 2023;4(4):515–524. 10.34067/KID.0000000000000074 PMC10278839 36763793

[6] Tian X Kim JJ Monkley SM, . Podocyte-associated talin1 is critical for glomerular filtration barrier maintenance. J Clin Invest. 2014;124(3):1098–1113. doi:10.1172/jci69778 24531545 PMC3934159

[7] Sever S Schiffer M. Actin dynamics at focal adhesions: a common endpoint and putative therapeutic target for proteinuric kidney diseases. Kidney Int. 2018;93(6):1298–1307. doi:10.1016/j.kint.2017.12.028 29678354 PMC5967993

[8] Nguyen TN Uemura A Shih W Yamada S. Zyxin-mediated actin assembly is required for efficient wound closure. J Biol Chem. 2010;285(46):35439–35445. doi:10.1074/jbc.m110.119487 20801875 PMC2975167

[9] Smith MA Blankman E Gardel ML Luettjohann L Waterman CM Beckerle MC. A zyxin-mediated mechanism for actin stress fiber maintenance and repair. Dev Cell. 2010;19(3):365–376. doi:10.1016/j.devcel.2010.08.008 20833360 PMC2954498

[10] Tan R Patni H Tandon P, . Nef interaction with actin compromises human podocyte actin cytoskeletal integrity. Exp Mol Pathol. 2013;94(1):51–57. doi:10.1016/j.yexmp.2012.06.001 22721673 PMC3463768

[11] Sealfon R Mariani L Avila-Casado C, . Molecular characterization of membranous nephropathy. J Am Soc Nephrol. 2022;33(6):1208–1221. doi:10.1681/ASN.2021060784 35477557 PMC9161788

[12] Zambrano S He L Kano T, . Molecular insights into the early stage of glomerular injury in IgA nephropathy using single-cell RNA sequencing. Kidney Int. 2022;101(4):752–765. doi:10.1016/j.kint.2021.12.011 34968552

[13] Latt KZ Heymann J Jessee JH, . Urine single-cell RNA sequencing in focal segmental glomerulosclerosis reveals inflammatory signatures. Kidney Int Rep. 2022;7(2):289–304. doi:10.1016/j.ekir.2021.11.005 35155868 PMC8821042

[14] Abedini A Zhu YO Chatterjee S, . Urinary single-cell profiling captures the cellular diversity of the kidney. J Am Soc Nephrol. 2021;32(3):614–627. doi:10.1681/ASN.2020050757 33531352 PMC7920183

[15] Luecken MD Theis FJ. Current best practices in single-cell RNA-seq analysis: a tutorial. Mol Syst Biol. 2019;15(6):e8746. doi:10.15252/msb.20188746 31217225 PMC6582955

